# Array2BIO: from microarray expression data to functional annotation of co-regulated genes

**DOI:** 10.1186/1471-2105-7-307

**Published:** 2006-06-16

**Authors:** Gabriela G Loots, Patrick SG Chain, Shalini Mabery, Amy Rasley, Emilio Garcia, Ivan Ovcharenko

**Affiliations:** 1 Biosciences Directorate, Lawrence Livermore National Laboratory, Livermore, California 94550, USA; 2 Computational Directorate, Lawrence Livermore National Laboratory, Livermore, California 94550, USA; 3 Department of Energy Joint Genome Institute, Walnut Creek, CA 94598, USA

## Abstract

**Background:**

There are several isolated tools for partial analysis of microarray expression data. To provide an integrative, easy-to-use and automated toolkit for the analysis of Affymetrix microarray expression data we have developed Array2BIO, an application that couples several analytical methods into a single web based utility.

**Results:**

Array2BIO converts raw intensities into probe expression values, automatically maps those to genes, and subsequently identifies groups of co-expressed genes using two complementary approaches: (1) comparative analysis of signal versus control and (2) clustering analysis of gene expression across different conditions. The identified genes are assigned to functional categories based on Gene Ontology classification and KEGG protein interaction pathways. Array2BIO reliably handles low-expressor genes and provides a set of statistical methods for quantifying expression levels, including Benjamini-Hochberg and Bonferroni multiple testing corrections. An automated interface with the ECR Browser provides evolutionary conservation analysis for the identified gene loci while the interconnection with Crème allows prediction of gene regulatory elements that underlie observed expression patterns.

**Conclusion:**

We have developed Array2BIO – a web based tool for rapid comprehensive analysis of Affymetrix microarray expression data, which also allows users to link expression data to Dcode.org comparative genomics tools and integrates a system for translating co-expression data into mechanisms of gene co-regulation. Array2BIO is publicly available at

## Background

Microarray experiments provide a rapid method for directly profiling the expression pattern of an entire gene repertoire in a genome. This experimental approach has become routine for the *en masse *identification of genes associated with different biological processes. We have developed a multifunctional, user-friendly, web-interactive microarray analysis tool, Array2BIO, that identifies and functionally characterizes co-expressed genes. In addition, it integrates other genomic, transcriptional and gene regulatory tools (Loots and Ovcharenko 2005) to allow scientists to explore mechanisms of gene co-regulation specific to co-functional groups of genes. Array2BIO permits users to functionally characterize clusters of co-expressed genes, identify putative biological activities, study interaction networks, as well as predict modules of transcription factors regulating eukaryotic gene expression in different tissues and under different conditions.

## Implementation

### Microarray data analysis

#### Background correction

Array2BIO follows the original Affymetrix procedure of background correction. An array of probes is separated into 16 zones (4 × 4 grid). Raw intensities for each zone are ranked and the background level is defined as the 2% lowest intensity for each zone. The distance from each probe to the zone center is used to estimate the background level at each probe location, which is then subtracted from the raw probe intensity.

#### Filtering out non-specific hybridization

Each probe intensity is measured in duplicates – a perfect match (PM) intensity and mismatch (MM) intensity, where the MM intensity estimates the cross-reactivity with other genes. Array2BIO excludes all probes with a PM intensity less than 1.25*MM. It also calculates the ratio of probes with specific hybridization that pass through this filtering. MM intensity is subtracted from the PM intensity for the remaining probes, such that the raw intensity is measured as the relative (PM-MM) intensity.

#### Normalization and Log_2 _transformation

Median (PM-MM) array intensity I˜
 MathType@MTEF@5@5@+=feaafiart1ev1aaatCvAUfKttLearuWrP9MDH5MBPbIqV92AaeXatLxBI9gBaebbnrfifHhDYfgasaacH8akY=wiFfYdH8Gipec8Eeeu0xXdbba9frFj0=OqFfea0dXdd9vqai=hGuQ8kuc9pgc9s8qqaq=dirpe0xb9q8qiLsFr0=vr0=vr0dc8meaabaqaciaacaGaaeqabaqabeGadaaakeaacuWGjbqsgaacaaaa@2DD6@ is calculated for the remaining probes after the filtering step. Individual (PM-MM) probe intensities *I*_*i *_undergo normalization and a base 2 logarithmic transformation:

*EP*_*i *_= log_*s*_(*I*_*i*_/I˜
 MathType@MTEF@5@5@+=feaafiart1ev1aaatCvAUfKttLearuWrP9MDH5MBPbIqV92AaeXatLxBI9gBaebbnrfifHhDYfgasaacH8akY=wiFfYdH8Gipec8Eeeu0xXdbba9frFj0=OqFfea0dXdd9vqai=hGuQ8kuc9pgc9s8qqaq=dirpe0xb9q8qiLsFr0=vr0=vr0dc8meaabaqaciaacaGaaeqabaqabeGadaaakeaacuWGjbqsgaacaaaa@2DD6@).

#### Probe to tag mapping

Affymetrix *.CDF *files are used to map individual probe intensities *EP*_*i *_onto Affymetrix gene tags *GP*_*j*_. Usually each tag accumulates ~ 10 good probes that span the corresponding gene transcript.

#### Averaging experiment replicas

Several experimental replicas can be averaged in comparative analysis to reliably estimate signal and background gene expression levels.

#### Filtering out the outliers

It is common to observe that the expression level of several gene probes differs significantly from the median level of transcript expression G˜
 MathType@MTEF@5@5@+=feaafiart1ev1aaatCvAUfKttLearuWrP9MDH5MBPbIqV92AaeXatLxBI9gBaebbnrfifHhDYfgasaacH8akY=wiFfYdH8Gipec8Eeeu0xXdbba9frFj0=OqFfea0dXdd9vqai=hGuQ8kuc9pgc9s8qqaq=dirpe0xb9q8qiLsFr0=vr0=vr0dc8meaabaqaciaacaGaaeqabaqabeGadaaakeaacuWGhbWrgaacaaaa@2DD2@*P*_*j *_. To filter out the outliers, Array2BIO excludes transcript probes with expression values that differ from G˜
 MathType@MTEF@5@5@+=feaafiart1ev1aaatCvAUfKttLearuWrP9MDH5MBPbIqV92AaeXatLxBI9gBaebbnrfifHhDYfgasaacH8akY=wiFfYdH8Gipec8Eeeu0xXdbba9frFj0=OqFfea0dXdd9vqai=hGuQ8kuc9pgc9s8qqaq=dirpe0xb9q8qiLsFr0=vr0=vr0dc8meaabaqaciaacaGaaeqabaqabeGadaaakeaacuWGhbWrgaacaaaa@2DD2@*P*_*j *_by an *x *number of standard deviations *σ*_*j *_(thresholds defined by the user). A strict filtering (1* *σ*_*j*_) and a medium stringency filtering (2* *σ*_*j*_) are set as defaults for the comparative and clustering analyses, correspondingly.

### Statistical methods (comparative analysis)

#### Handling low-expressors

The significance of fold-difference in intensity values (ie. expression) varies dramatically for low- vs. high-expressor genes. This occurs because dividing a small number by another small number (in case of low-expressors) can result in a large fold-difference simply by chance. Array2BIO utilizes local mean normalization and local variance correction across intensities to differentially handle low- and high-expressors and to define separate fold-difference thresholds for different intensity levels. Array2BIO employs an approach highly similar to the previously described SNOMAD method (Colantuoni et al. 2002) and represents a 'pooled local variance' approach with 100 bins of gene tags. First, fold-expression levels of Affymetrix tags are ordered by their average expression level across signal and control data. Then gene tags are binned into 100 groups by the average expression level and local variation of fold-expressions is calculated for each group. This allows one to compute the local standard deviation (*σ*^*i*^) and subsequently local z-score (*z*_*j*_) of fold-difference for each individual gene tag in each *i*-th group that *j*-th gene tag belongs to:

zj=Xj−Xi¯σi
 MathType@MTEF@5@5@+=feaafiart1ev1aaatCvAUfKttLearuWrP9MDH5MBPbIqV92AaeXatLxBI9gBaebbnrfifHhDYfgasaacH8akY=wiFfYdH8Gipec8Eeeu0xXdbba9frFj0=OqFfea0dXdd9vqai=hGuQ8kuc9pgc9s8qqaq=dirpe0xb9q8qiLsFr0=vr0=vr0dc8meaabaqaciaacaGaaeqabaqabeGadaaakeaacqWG6bGEdaWgaaWcbaGaemOAaOgabeaakiabg2da9maalaaabaGaemiwaG1aaSbaaSqaaiabdQgaQbqabaGccqGHsisldaqdaaqaaiabdIfaynaaCaaaleqabaGaemyAaKgaaaaaaOqaaGGaciab=n8aZnaaCaaaleqabaacbiGae4xAaKgaaaaaaaa@3ABF@, where Xi¯
 MathType@MTEF@5@5@+=feaafiart1ev1aaatCvAUfKttLearuWrP9MDH5MBPbIqV92AaeXatLxBI9gBaebbnrfifHhDYfgasaacH8akY=wiFfYdH8Gipec8Eeeu0xXdbba9frFj0=OqFfea0dXdd9vqai=hGuQ8kuc9pgc9s8qqaq=dirpe0xb9q8qiLsFr0=vr0=vr0dc8meaabaqaciaacaGaaeqabaqabeGadaaakeaadaqdaaqaaiabdIfaynaaCaaaleqabaGaemyAaKgaaaaaaaa@2F7E@ is the average fold-difference in expression of the *i*-th group. Differentially expressed tags identified by Z-score greater than 2.0 are selected for further analysis (Figure 3).

#### Welch's t-test of differential expression significance

Signal and control tags that survive the balance analysis of low- and high-expressors are next subjected to statistical testing using the Welch's t-test method. Statistical testing is performed on the average signal and control tag expression using standard deviations of their probe expression distribution. A p-value is assigned to every differentially expressed tag and tags with p-values less than 0.05 are selected for multiple testing correction analyses.

#### Mapping Affymetrix tags onto UCSC known genes

Array2BIO first identifies a set of unique (non-overlapping) genes in a genome matching the original.*CEL *file by using the 'known genes' annotation provided by the UCSC Genome Browser database (Karolchik et al. 2003). Next, Affymetrix tags are mapped onto (and are grouped by) UCSC 'known genes'. Accession numbers for the corresponding mRNA sequences and their genomic locations are retrieved for each gene during the mapping process. This information is next used to dynamically link genes to the NCBI database and to the ECR Browser.

#### Gene Ontology (GO) and KEGG analyses of biological functions and gene interactions

Array2BIO utilizes a locally installed version of the Gene Ontology (GO) (Harris et al. 2004) and KEGG (Ogata et al. 1999) databases to contrast the distribution of differentially expressed functional categories of genes to the average distribution in the corresponding genome. Observed and expected category population values are compared and the statistical 'enrichment' (or 'depletion') of a category is quantified by using hypergeometric distribution statistics. Functional categories with p-values smaller than 0.05 are selected for subsequent multiple testing correction analyses. The GO database provides biological classification of gene function through membership to functional categories that relate to certain biological processes, molecular functions, or to cellular components. The KEGG database combines information on gene interactions that are grouped into (1) metabolism, (2) genetic information processing, (3) environmental information processing, (4) cellular processes, and (5) human diseases categories.

#### Correction for multiple testing

Array2BIO performs correction for multiple testing to exclude false positive predictions associated with the statistical testing of differential tag expression or enrichment/depletion in GO and KEGG categories that is performed multiple times. Array2BIO provides two statistical methods to correct for multiple testing and also allows omitting multiple testing if the user does not want to apply this function. The default method used by Array2BIO is the medium stringency Benjamini-Hochberg correction (Benjamini and Hochberg 1995). Benjamini-Hochberg correction is based on controlling the false discovery rate (FDR) – the expected proportion of false discoveries amongst the rejected hypothesis. In general it provides a good balance between discovery of statistically significant differences and limitation of false positive occurrences. Alternatively, the Bonferroni correction method can be applied. The latter is one of the most stringent multiple testing correction methods and can be used to select for the most outstanding overexpressor genes or enriched/depleted functional categories.

### Clustering analysis

#### Microarray data clustering

Array2BIO utilizes the Unix version of the Cluster tool (Eisen et al. 1998). Cluster's hierarchical analysis is implemented into Array2BIO, which allows clustering of genes and/or conditions; provides 9 distance measures and 4 methods. Due to Cluster limitations, Array2BIO restricts the maximum number of clustered transcripts to less than 2500 genes. Genes are ranked by their standard deviation in expression across different conditions. Genes with the largest variation from their average expression across all conditions are selected for clustering.

#### Interactive tree visualization

Array2BIO provides an interactive web utility for visualizing clustering results, which is similar in graphical display and operation to Java TreeView (Saldanha 2004). Clustered gene expression across multiple conditions is visualized in a matrix format. The tree of clustering relationships is given to the left of the gene expression image (Figure 4A). A mouse click on a tree branch generates a 'zoom in' image of that branch and gives a detailed description of related genes (including gene names, accession numbers, corresponding Affymetrix tags, and genomic locations) (Figure 4B).

### Interconnection with external tools

#### ECR Browser – evolutionary conservation analysis

The ECR Browser (Ovcharenko et al. 2004) is a dynamic whole-genome navigation tool for visualizing and studying evolutionary relationships among genomes. Evolutionary Conserved Regions (ECRs) are extracted from genome alignments, mapped to genomes, and graphically visualized in relation to the genes that have been annotated in the reference genome.

#### Creme 2.0 – identification of clusters of transcription factor binding sites in promoters

Crème 2.0 (Sharan et al. 2004) relies on a database of putative transcription factor binding sites that have been carefully annotated across the human genome using evolutionary conservation with the mouse and rat genomes. An efficient search algorithm is applied to this data set to identify combinations of transcription factors whose binding sites tend to co-occur in close proximity to the start site of the input gene set. These combinations are statistically evaluated, and significant combinations are reported and visualized.

#### NCBI – detailed sequence information

Detailed mRNA transcript information including: nucleotide and protein sequences, related publications, gene annotation, etc. are provided through the dynamic interconnection to the NCBI database.

## Results and discussion

**Figure 1:**
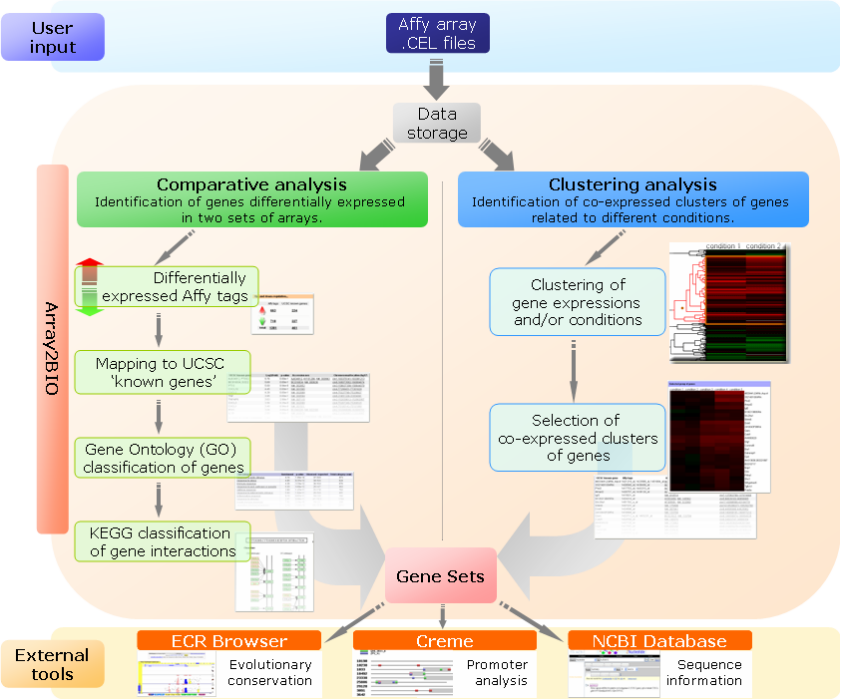
Schematic flowchart of the Array2BIO analysis.

Figure 1 summarizes the schematics behind Array2BIO analysis. Users are required to submit input textual. CEL files – (i.e. the standard output data derived from Affymetrix microarray experiments). Array2BIO performs multi-step data analysis and filtering, including background correction, exclusion of non-specific hybridizing probes, normalization and logarithmic transformation of raw intensities. Individual probes are automatically mapped to Affymetrix tags and subsequently to UCSC 'known genes' (Karolchik et al. 2003). In contrast to other available microarray analysis software, Array2BIO analysis also incorporates a balanced analysis of low- and high-expressor genes thus providing a reliable method for handling low-expressors that would otherwise lead to false positive predictions.

Two complementary methods of microarray data analysis are incorporated into the Array2BIO software: 1) comparative and 2) clustering analyses. Comparative analysis identifies genes that are differentially regulated in reference to a control sample (for example gene expression in transgenic animals compared to non-transgenic, wild-type littermates). Clustering analysis identifies groups of genes that are co-expressed under different experimental conditions (e.g. when analyzing time-course experiments).

The automated functional classification of co-expressed genes is based on the Gene Ontology (Harris et al. 2004) database and allows the identification of 'enriched' or 'depleted' categories in assigned biological processes, molecular functions, and cellular components. Integrated KEGG (Ogata et al. 1999) classification of gene interactions identifies major biochemical processes that underlie observed differences in gene expression and groups genes into five main categories – (1) metabolism, (2) genetic information processing, (3) environmental information processing, (4) cellular processes, and (5) human diseases.

Every group of differentially expressed genes identified using Array2BIO is dynamically linked to the Evolutionary Conserved Region (ECR) Browser (Ovcharenko et al. 2004) and to the Cis-REgulatory Module Explorer tool (Sharan et al. 2004), as well as to the NCBI database. The ECR Browser provides multi-species evolutionary conservation information for individual genes, and the NCBI database provides detailed information about mRNA sequences and related proteins. The Crème 2.0 tool allows the user to perform an additional step to functionally annotate groups of human genes through the analysis of their promoter elements. In this process the tool will identify shared clusters of evolutionary conserved transcription factor binding sites within promoters of co-expressed genes. Combined, these tools provide a wealth of information regarding the gene(s) in question, its conservation, its transcripts, as well as candidate regulatory mechanisms underlying the observed transcriptional response from the microarray data.

### Application to the analysis of host-pathogen interactions

To illustrate the different levels of information that can be obtained from Array2Bio analysis we have processed microarray expression data generated in a time-course experiment of human cells infected with *Yersinia pestis*. The plague (commonly known as the Black Death) is an infectious disease that has devastated much of the known world in the 14^th ^century, and killed more than 200 million people during three major pandemics. It is primarily a disease in rodents caused by an infection with the bacterium *Yersinia pestis*, but can be transmitted to humans through the bite of infected fleas.

**Figure 2:**
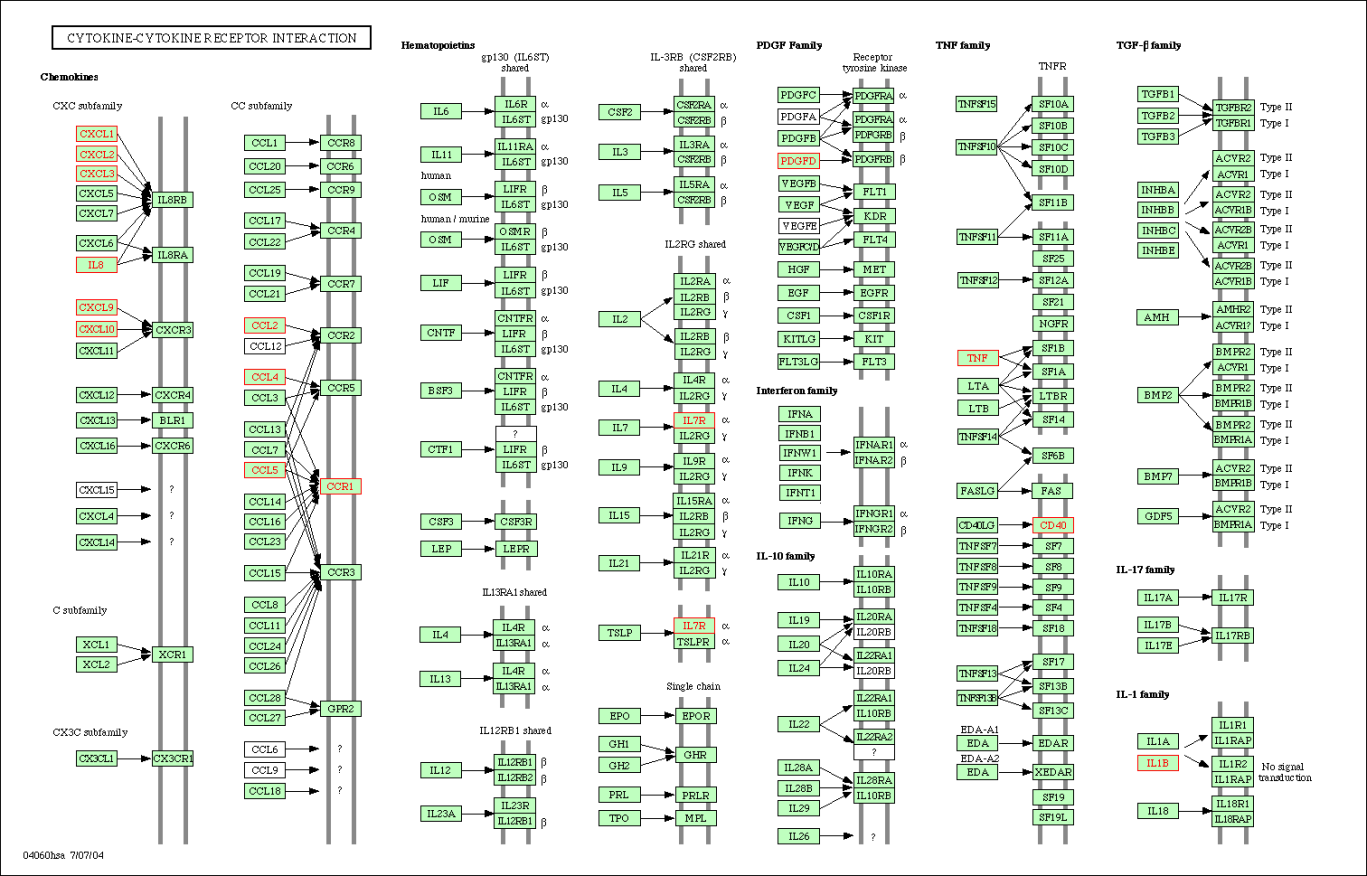
Array2BIO automatically fetches KEGG maps (Ogata et al. 1999) from the KEGG web site and utilizes locally generated data to color-demarcate individual genes. KEGG snapshot of cytokine-cytokine receptor interactions that are related to the *Y. pestis *infection with identified genes in red.

**Figure 3:**
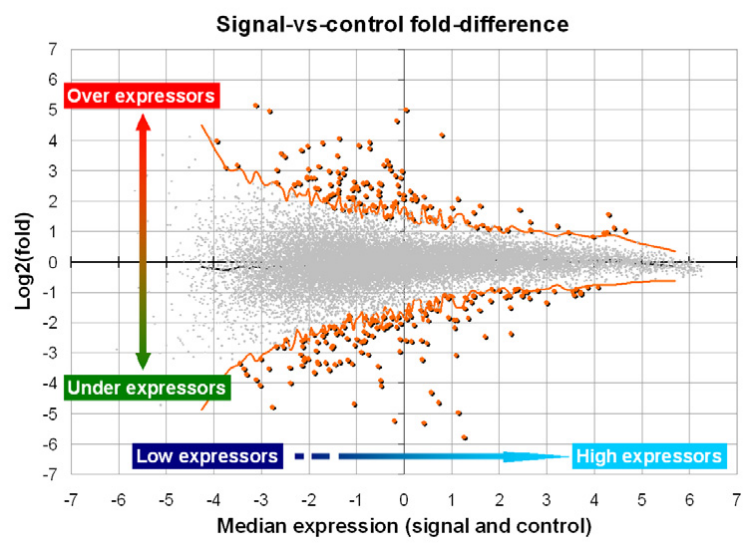
SNOMAD local Z-test for handling low-expressors. Signal versus control fold different in expression is plotted against the median signal and control expression. Orange dots represent over- and under-expressors.

**Figure 4:**
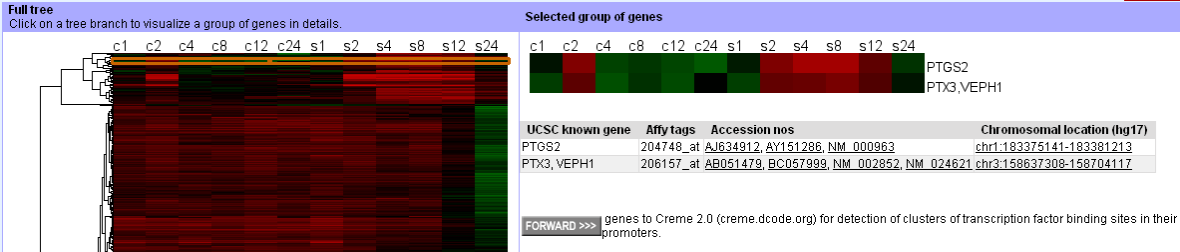
Visualization of clustering analysis. A full clustering tree across 5 control (cN) and 5 signal (sN) conditions (A) and a *zoom in *into two genes (B). The *zoom in *function is performed by clicking on a region in the full clustering tree, in this case, depicted by the orange frame.

To address host-pathogen interactions and elucidate the molecular mechanisms underlying the virulence of this pathogen during human infection, human dendritic cells were exposed to *Y. pestis *infection, and RNA samples were collected at different time points and gene expression was analyzed by microarrays. Using Array2Bio we compared HG-U133A microarray expression data of human dendritic cells at 4 hours after exposureto *Y. pestis *to mock-exposed cells. We observed significant increases and decreases in expression (as measured using the Welch's t-test analysis with Benjamini-Hochberg correction for multiple testing) for 139 and 81 human genes, respectively. Gene Ontology (GO) analysis identified 31 'enriched' biological processes and 5 molecular functions corresponding to up-regulated genes; while none were found for down-regulated genes. As expected, the majority of these categories were related to the human immune response, including the "response to pest, pathogen or parasite" (Table 1). The chemokine (cytokines with chemotactic activities) category was ~ 20-fold 'enriched' when compared to the expected values due to chance alone. Eighteen percent of all human chemokines (primarily CXC chemokines) are activated in response to *Y. pestis *invasion. KEGG analysis of the corresponding gene interactions identified a family of up-regulated CXC cytokines acting upstream of the IL8RB receptor, and several other receptor genes (Figure 2). These pathways are likely to reflect the core response of human dendritic cells to this infection. KEGG analysis of enriched cellular processes highlighted two related subcategories: (1) apoptosis (p < 0.001) and (2) cell growth and death (p < 0.002). Six genes are shared between these two subcategories and may be key players in the etiology of this infectious disease.

We performed Crème 2.0 analysis on 25 genes identified in this study that are related to the "response to pest, pathogen or parasite" GO category. Crème 2.0 predicted transcription factors that potentially act as key regulators of these genes and are likely to up-regulate their expression during *Y. pestis *infection. Several transcription factors binding sites conserved between human and rodents were significantly enriched in the promoters of these genes, including several members of the STAT and NFKB families, as well as TATA transcription factors. While the TATA transcription factor plays a basal role in the TATA-box recognition, the two other identified transcription factor families are known to be involved in regulating the immune system. STAT and NFKB proteins respond to cytokines, are associated with inflammatory disease and can lead to inappropriate immune cell development. (Hirayama et al. 2005; O'Shea et al. 2005).

## Conclusion

Array2BIO is an addition to the Dcode.org collection of tools (Loots and Ovcharenko 2005) that permits the efficient and unique integration of comparative and transcriptional regulatory genomic utilities with a multi-functional framework for analyzing gene expression data. Most importantly, Array2BIO represents a web-based tool/utility for integrative analysis of microarray expression data that permits experimental biologists with limited background in statistics to perform detailed, highly informative analysis comparable to sophisticated software packages catered to the expert statistician. A "single-click" implementation of the variety of biological characterizations into a single tool permits the standardized, prompt identification of co-expressed genes, their functional annotation, the identification of related interaction pathways, and prediction of key transcription factors underlying observed gene expression responses. Currently our server provides 200 Mb of disk space per account. All the input CEL files are compressed allowing users to store over one hundred CEL files per account. We anticipate additional disk space to be made available per account, with each new release of the tool.

## Availability and requirements

Project name: Array2BIO;

Project home page: ;

Operating system(s): Web-based, platform independent;

Programming language: PHP;

License: There are no access restrictions and no need for a license for both academic and private entities to use this research tool.

## Authors' contributions

GGL participated in designing the scheme of the tool and writing the manuscript. PSGC, SM, AR, and EG carried out experimental studies. IO coordinated the developments, created the tool and drafted the manuscript.

## Figures and Tables

**Table 1:** Five of the most overrepresented GO biological processes and molecular functions that corresponding to *Yersinia pestis *infection.

**Biological processes**	**Enrichment**	**p-value**	**Observed/expected**	**Total category count**
response to biotic stimulus	4.16	1.85e-14	37/8.9	873
response to stress	4.05	6.37e-13	34/8.4	824
immune response	4.36	3.33e-12	30/6.9	676
response to pest, pathogen or parasite	5.30	4.48e-12	25/4.7	463
defense response	3.99	1.27e-11	31/7.8	762

				

**Molecular functions**	**Enrichment**	**p-value**	**Observed/expected**	**Total category count**

chemokine activity	17.07	1.72e-8	8/0.5	46
chemokine receptor binding	17.07	1.72e-8	8/0.5	46
G-protein-coupled receptor binding	15.40	4.01e-8	8/0.5	51
cytokine activity	5.27	7.35e-6	11/2.1	205
transcription factor activity	2.48	2.92e-4	18/7.3	712
